# A regional systems intervention for suicide prevention in the Netherlands (SUPREMOCOL): study protocol with a stepped wedge trial design

**DOI:** 10.1186/s12888-019-2342-x

**Published:** 2019-11-19

**Authors:** Emma Hofstra, Iman Elfeddali, Margot Metz, Marjan Bakker, Jacobus J. de Jong, Chijs van Nieuwenhuizen, Christina M. van der Feltz-Cornelis

**Affiliations:** 1 Specialist Mental Health Institution, GGz Breburg, Tilburg, Netherlands; 2 Tranzo-Scientific Center for Care and Wellbeing, Tilburg University, Tilburg, Netherlands; 3 Department of Methodology and Statistics, Tilburg University, Tilburg, Netherlands; 4 Institute for Mental Health Care, GGzE, Eindhoven, Netherlands; 5 Mental Health and Addiction Research Group, Department of Health Sciences, Hull York Medical School, University of York, York, UK

**Keywords:** SUPREMOCOL, Suicide prevention, Systems intervention, Stepped wedge trial design, Study protocol, Collaborative care, Monitoring, Decision aid

## Abstract

**Background:**

In the Netherlands, suicide rates showed a sharp incline and this pertains particularly to the province of Noord-Brabant, one of the southern provinces in the Netherlands. This calls for a regional suicide prevention effort.

**Methods/design:**

Study protocol. A regional suicide prevention systems intervention is implemented and evaluated by a stepped wedge trial design in five specialist mental health institutions and their adherent chain partners. Our system intervention is called SUPREMOCOL, which stands for Suicide Prevention by Monitoring and Collaborative Care, and focuses on four pillars: 1) recognition of people at risk for suicide by the development and implementation of a monitoring system with decision aid, 2) swift access to specialist care of people at risk, 3) positioning nurse care managers for collaborative care case management, and 4) 12 months telephone follow up. Eligible patients are persons attempting suicide or expressing suicidal ideation. Primary outcome is number of completed suicides, as reported by Statistics Netherlands and regional Public Health Institutes. Secondary outcome is number of attempted suicides, as reported by the regional ambulance transport and police. Suicidal ideation of persons registered in the monitoring system will, be assessed by the PHQ-9 and SIDAS questionnaires at baseline and 3, 6, 9 and 12 months after registration, and used as exploratory process measure. The impact of the intervention will be evaluated by means of the RE-AIM dimensions reach, efficacy, adoption, implementation, and maintenance. Intervention integrity will be assessed and taken into account in the analysis.

**Discussion:**

The present manuscript presents the design and development of the SUPREMOCOL study. The ultimate goal is to lower the completed suicides rate by 20%, compared to the control period and compared to other provinces in the Netherlands. Moreover, our goal is to provide specialist mental health institutions and chain partners with a sustainable and adoptable intervention for suicide prevention.

**Trial registration:**

Netherlands Trial Register under registration number NL6935 (5 April 2018). This is the first version of the study protocol (September 2019).

## Background

Completed suicides and suicide attempts c physical and emotional harm on the individual. Significant others, the community, and even entire nations suffer the consequences of such tragic events [1, 2]. Annually, more than 800,000 suicides occur worldwide, of which over 56,000 were reported in the European Union (EU), and almost 2000 in the Netherlands [1, 3, 4]. Although suicide rates in the Netherlands were equal to the EU-average [3], they did show a sharp incline of 37% between 2007 and 2013 [4]. This might be related to the economic recession in 2008 and rigorous budget cuts in the mental health sector [5, 6]. Since 2013, the relative suicide rate in the Netherlands has been stable, without decreasing [4]. In Noord-Brabant, one of the twelve provinces of the Netherlands, this stabilization in the suicide rates did not take place, as the absolute number of suicide in this province increased by 64%. Remarkably, 30% of this 64% increase in completed suicides happened in the years after 2013 [4]. At the start of the grant application for this study in 2015, Noord-Brabant even ranked second nationally over the five previous years (2010–2014) and there were 293 suicides in 2014, which is 11,5 suicides per 100,000 residents [4]. This poses a problem in the Netherlands and more specifically a regional problem in Noord-Brabant.

An important issue in effective suicide prevention is that approximately two-thirds of suicide victims were not receiving mental health care [7, 8], while they were probably in need of it, as suicide occurs mostly in the context of mental disorders [1, 2]. This might be due to a lack of visibility of people at risk, as help-seeking behaviour for suicidality is low, possibly due to stigma and poor suicide literacy [9]. Moreover, transitions of and discharges from care are associated with an increased risk of suicide attempt or death [10, 11]. An exploration amongst stakeholders identified that a tentative explanation might be a lack of communication between health care providers of different institutions or lack of swift entry into specialist care due to logistical barriers and waiting lists.

Another problem concerns the identification of people at risk for suicide since there are many risk factors known that are also very common [2]. Suicide attempts and suicidal ideation are among the strongest predictors of completed suicide [1, 12]. Suicide risk is also elevated in case of job and financial problems, unbearable mental pain, lack of a support system, trauma, stigma, impulsive aggression, hopelessness, living alone, and being faced with loss [1, 2, 13]. In addition, suicide occurs more often in males than in females [1]. The predictive power of these individual risk factors is thus very low [2, 14]. There is yet no single tool, questionnaire or instrument that can predict suicide [15]. Clinical assessment can also be very hard, given the fact that about 45% of patients who died by suicide did meet with a primary care provider in the preceding month [7]. A large (*N* = 4800) longitudinal study found that prediction failed mostly, as 60% of patients that died by suicide had been categorized before as low risk by mental health professionals [16]. In the Netherlands, a Multidisciplinary Guideline Diagnostics and Treatment of Suicidal Behaviour (MGSB) was developed in 2012 [17]. A subsequent study aimed at training professionals for the assessment and treatment of suicidal behaviour -as recommended in this MGSB- resulted in greater guideline adherence, but did not result in lower suicide rates in the Netherlands so far [18].

This urgently calls for a suicide prevention effort in Noord-Brabant, aiming to reduce suicide by identifying people at risk for suicide and by a collaborative effort to improve the delivery of services by all relevant stakeholders.

## Rationale

This study considers completed suicides as mostly preventable deaths and in that vein will follow an example of a regional systems intervention study of preventable deaths in traffic trauma-related mortality in Orange County, USA [19]. A regional network of Specialty Mental Health Institutions, general hospitals, general practices, public health partners and community partners (schools, railway services, municipalities, agricultural organizations) will be implemented with the aim to diminish preventable deaths by suicide in Noord-Brabant. Our suicide prevention system is called SUPREMOCOL, which stands for Suicide Prevention by Monitoring and Collaborative Care, and is based on four pillars: [1] recognition of people at risk for suicide development and implementation of a monitoring system with decision aid, [2] swift access to specialist care of people at risk, [3] positioning nurse care managers for collaborative care case management, [4] and 12 months telephone follow up.

## Objectives

The aim of the present paper is to describe the content of the SUPREMOCOL regional systems intervention and the study design for the scientific evaluation. The four pillars of the intervention are described in the paragraph ‘Intervention’ and the hypothesis for the scientific study in the paragraph ‘Scientific evaluation’. The objective of the SUPREMOCOL project is to lower suicide rates in the province of Noord-Brabant, the Netherlands, by 20%. To this end, three sub-objectives are essential:

1. Establishing the SUPREMOCOL systems intervention, for persons at risk for suicide by improving delivery of services by a well-functioning chain of care on multiple levels, set up with the purpose to remain available after ending of the study.
2. Evaluate the effect of this multilevel suicide prevention systems intervention in terms of completed suicides and non-fatal suicide attempts in an embedded evaluation.
3. Explore the public health impact of the suicide prevention intervention by the RE-AIM framework, including which factors (i.e. patient or transition factors) are associated with early withdrawal of treatment, taking the relevant stakeholders i.e. professionals, patients and their significant others into account.

## Methods/design

### Intervention

#### Framework

In the development of our suicide prevention intervention we were inspired by a successful systems intervention, that aimed to improve access to care by the regionalization of trauma care for preventable traffic trauma-related deaths in Orange County, California [20]. A combination of swift triage and entrance to designated expert trauma care led to a decrease to one-eighth of the previous rate of preventable deaths, i.e. deaths that could have been prevented by better delivery of services [21]. Similar effects were found in several other regions implementing the same systems intervention [22]. Several key components of the intervention of West and colleagues [23, 24], were translated to our specific target group and context. One component is to work with the concept of preventable death; as in the case of traffic trauma, not all suicides may be preventable. This can, for example, be the case in people experiencing unbearable suffering and suicidality due to severe mental disorders combined with debilitating somatic illness. For our systems intervention, we deemed suicides preventable if they could be prevented by better delivery of services. For example, if someone experiences suicidality due to a psychiatric disorder which has been intensively -but yet unsuccessfully- treated in primary care, a completed suicide might be prevented by swiftly transferring the person to specialist mental health care. This view has been advocated by Wasserman et al. (2016), who coined a completed suicide with the term unnecessary death [25]. Another component is to do first triage on the spot by field professionals if indeed a preventable death is the case. Moreover, providing trauma treatment, not at the closest emergency room, but at the emergency room where an experienced trauma team is available is also an important component. This has been proven to be more effective despite the initial time loss outside the hospital, due to the high efficiency in the trauma specialist center, once the patient arrives there [20]. Establishing swift access to specialized trauma care in the institution itself in a practical manner could also be translated into our approach. Our suicide prevention system is called SUPREMOCOL, which stands for Suicide Prevention by Monitoring and Collaborative Care, and focuses on four pillars.

### Pillar 1: development and implementation of a monitoring system with decision aid

The first pillar concerns developing and implementing a monitoring system with decision aid to support professionals in reporting, assessing and monitoring people at risk for suicide. By using this system, we expect to identify more people at risk for suicide and to provide a tool for suicide risk assessment. In the monitoring system, both (mental) health care and non-(mental) health care professionals are involved to signal people at risk and to refer them to the closest specialist mental health care institution (SMHI). A decision aid is built-in to support health care professionals in methodical/systematic, evidence-based suicide risk assessment. The decision aid for suicide risk assessment consists of two parts: the Patient Health Questionnaire-9 (PHQ-9) and an additional risk assessment tool that is developed by the project group. The PHQ-9 is a reliable and valid measure of depression severity and has been proven useful in both clinical and research practice [26]. In this study, the Dutch translation is used. The scores comprise the time period over the last 2 weeks and are 0 (not at all), 1 (several days), 2 (more than half of the days), and 3 (nearly every day) [26]. Item 9 of the PHQ-9 questionnaire had been found to be a robust predictor of suicide attempts and deaths, regardless of age [27], therefore, item 9 is used in this study as a first screener for suicidality. In this item, the person scores how often he/she is bothered by ‘Thoughts that you would be better off dead, or thoughts of hurting yourself in some way’ [26]. Although the whole PHQ-9 questionnaire will be filled out, suicide risk will be only calculated based on the score on item 9. If a person scores ‘0’ or ‘1’ on this item, suicide risk is considered as *low* risk, while the suicide risk is considered as a *medium* to *high* if a person scores ‘2’ or ‘3’. If the risk is medium or high, a second screener will follow to further estimate the suicide risk, which is the decision aid. This decision aid is developed by the project group and is based on seven questions for suicide risk which are answered with ‘yes’ or ‘no’ and is provided in Table 1.

**Table 1:** Decision aid

Domain	Question
In the past month	1. Did you have thoughts of being better off when you were dead or did you wish you were dead?
	2. Did you want to hurt yourself?
	3. Did you have thoughts about suicide?
	4. Did you make suicide plans?
	5. Did you attempt suicide?
In life	6. Did you have thoughts of being better off when you were dead or did you wish you were dead?
Clinical impression	7. Is there any acute danger in the behaviour of the person?

**Fig. 1:**
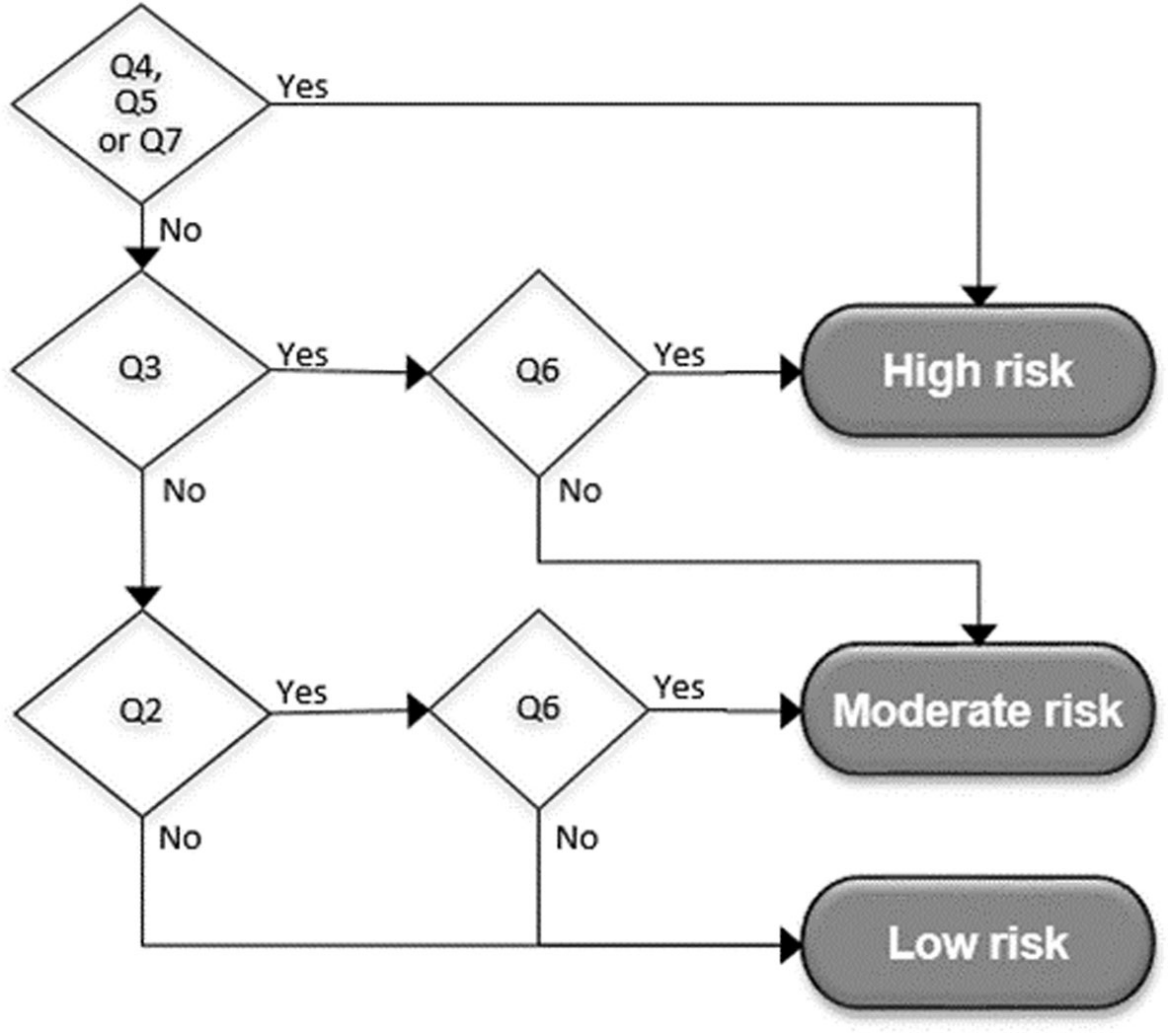
Algorithm of the decision aid

Based on the answers of the person, suicide risk is calculated, which can be either high, moderate or low. The suicide risk is estimated as *high* if the person answers ‘yes’ on question 4 or 5, or on both questions 3 and 6, or if question 7 is answered by the clinician with ‘yes’. If the person answers ‘yes’ only on question 3, or only on questions 2 and 6, the estimated risk is *moderate*. The suicide risk is otherwise estimated as *low* risk. This algorithm is visually displayed in Fig. 1. In the monitoring system, a cut-off is used, in which a moderate and high estimated risk are both labelled as ‘increased risk’.

Non-health care professionals do not perform a suicide risk assessment as they are not authorized to do so. When they signal a person at risk for suicide, the risk assessment will be performed in the SMHI, in the same manner. The monitoring system also provides follow-up care from the SMHI, which is described in pillar 2, 3 and 4.

### Pillar 2: providing swift access

Providing swift access to people registered in the monitoring system by specialist crisis teams of SMHIs is the second pillar of our intervention. The first time period after someone showed suicidal behavior remains very vulnerable for relapse, as suicide occurs mostly in people not receiving mental health care [7, 8] and in transitions of and discharges from care [10, 11]. But although suicide risk is thirty times higher in people with a previous attempt [1], one attempt may not necessarily be followed by another one, if proper mental health care is provided after the attempt. Suicide risk can also abate after a failed attempt. Out of 515 patients who attempted suicide at the Golden Gate Bridge, 94% did not die from suicide, at a 26 years follow-up [28]. Moreover, it has been found that appropriate pharmacological or psychotherapeutic treatment may prevent suicide [15, 29]. A recent systematic review and meta-analysis indicated that suicide prevention interventions were found to be effective in preventing completed suicides [30]. Providing swift access to people identified with suicidal behavior to specialist mental health care is thus highly desirable. In this study, swift access will be facilitated by the monitoring system and will be provided by the SMHI. In case a persons’ suicidality is not mainly caused by a psychiatric disorder, but for example, due to financial or somatic problems, the person might be referred to other (health) care settings if that is more appropriate.

### Pillar 3: positioning nurse care managers according to collaborative care

The third pillar of our intervention is the positioning of nurse care managers to collaborate with psychiatrists in assessment, case management, and guidance to treatment according to the collaborative care model. Collaborative care is an intensive care model that involves a number of health care professionals working together, such as a medical doctor, a case manager, and a psychiatrist. The case manager has regular contact with the person and organizes care, together with the medical doctor and specialist, and may offer help. There is ample evidence worldwide that collaborative care is successful in the treatment of depression [31–35]. Collaborative care has also been shown to be feasible, acceptable and effective in the prevention and reducing suicidal ideation [29]. A Cochrane review indicated that the implementation of treatment guidelines succeeds better if nurses are trained with that purpose [36]. In this study, collaborative care will be embedded in pillar two and four. Nurse care managers will collaborate with the person and his/her psychiatrist, clinical psychologist and general practitioner in suicide risk assessment, providing swift access to treatment, and monitoring. They will also signal potential problems in the continuity of care and will communicate this with the involved professionals.

### Pillar 4: providing one-year telephone monitoring

Providing telephone monitoring at five fixed times during 1 year to people registered in the monitoring system to enhance adherence to treatment is the fourth pillar of our intervention. The first year after a person showed suicidal behavior, usually many transitions within (mental) health care settings occur. The risk of suicide is increased during these transitions, as it has been found that of those who have died by suicide and were previously receiving mental health care, 24% were discharged in the previous 3 months. Most of these suicides already occurred in the first week after discharge [37]. Often there was non-compliance with treatment and loss of contact with services prior to the suicide [37]. This might be due to the lack of long-term monitoring follow up of people at risk for suicide. Multiple reviews already reported that structured follow-up contact with high-risk individuals, such as people that attempted suicide, decreased future suicidal behavior [11, 29]. Cebriá et al. (2013) found in a randomized controlled trial that providing patients at a general hospital emergency room that had attempted suicide with appropriate care and telephone follow up reduced the rate of patients reattempting suicide by 8%, compared to care as usual [38]. In our study, a one-year telephone follow-up care is provided to the people registered in the monitoring system. This follow-up aims to monitor the suicide risk and the continuation of care and is provided by the SMHI. The SMHI professional will signal any problems in the monitoring contact, and will communicate this with the clinician or general practitioner and will arrange new appropriate care if needed.

### Target population

The target population for the intervention are people that present themselves to, or are identified by, a professional of one of the participating institutions by showing signs of suicidal behavior. In this study, suicidal behavior includes both suicidal ideations and actions -preparatory and attempting- that are undertaken with the intention to die [17]. Inclusion criteria for registration in the monitoring system are 1) having a medium to high suicide risk according to the decision aid (see paragraph ‘Framework’ for the decision aid), 2) being a resident of the province of Noord-Brabant, and 3) giving permission to be registered in the monitoring system. People will be excluded if 1) low suicide risk is assessed, 2) they are not living in Noord-Brabant, and/or 3) they do not give consent to be registered in the monitoring system.

### Procedure of the intervention

The procedure of SUPREMOCOL is divided into three steps. In the first step, a person with suicide risk is registered in the monitoring system. A first contact with the crisis care manager is performed after registration, which is the second step in the procedure. In the third step, follow-up monitoring is provided to the people registered in the monitoring system. The four pillars of SUPREMOCOL are embedded in all steps.

### Step 1: registration in the monitoring system

In our systems intervention, both (mental) health care professionals - such as emergency room physicians, general practitioners or school psychologists - as well as non-health care professionals - such as railway professionals - can signal people at risk and register them in the monitoring system. When participating professionals signal people at risk, they first ask them for permission for registration in the monitoring system (according to the European Union General Data Protection Regulation [GDPR]). When permission for processing personal data is given, professionals who work as a (mental) health care professional perform a suicide risk assessment via the decision aid, which is built-in the monitoring system. The decision aid provides feedback based on the input provided by the persons’ answers on a questionnaire and observations made by the professional. If the suicide risk is *low* according to the decision aid, the person will not be registered and the professional is advised to refer the person to his/her general practitioner. If the suicide risk is *medium* or *high*, the person will be registered in the monitoring system, and their registration is automatically sent to the nearest SMHI, this is based on the postal code of the person who will be registered. Professionals that do not work in (mental) health care do not perform a suicide risk assessment as they are not authorized and trained to do so. They will register people in the monitoring system based on their own estimation. In these cases, the risk assessment will be performed later on by the SMHI crisis care professional who can use the decision aid to do so.

### Step 2: first contact with the crisis care professional

**Fig. 2:**
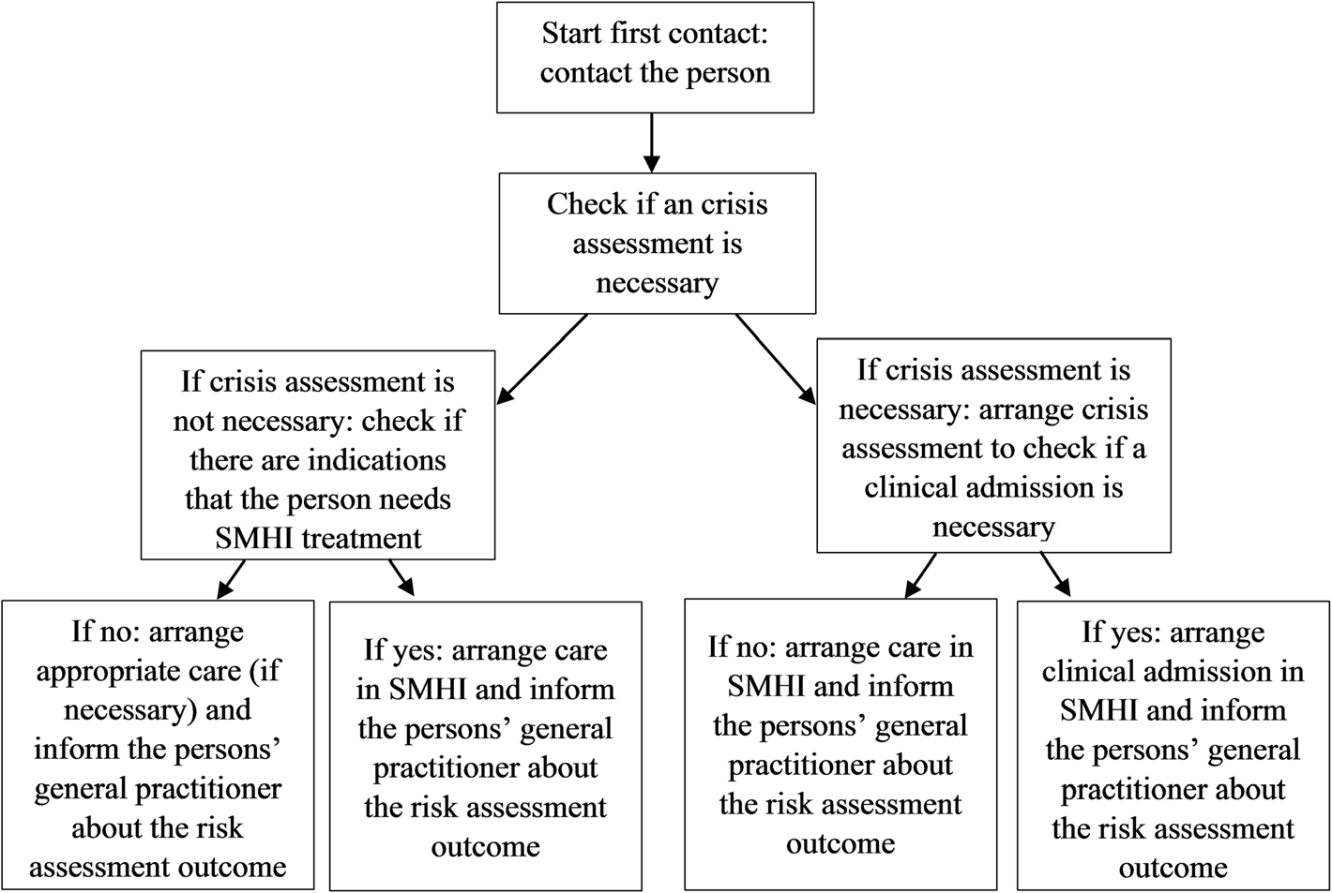
Flowchart of the first contact

The (daily) check for new enrolments in the monitoring system is conducted by crisis care professionals from the SMHI. This professional will actively seek contact on a daily basis with the people registered. They will check if a crisis assessment is necessary and they will ensure that these people receive swift access to appropriate care, most probably in the SMHI itself. SMHI professionals will work according to the collaborative care model by collaborating with the person and the involved professionals during 1 year. A flowchart of the first contact is provided in Fig. 2 .

### Step 3: follow-up monitoring

**Fig. 3:**
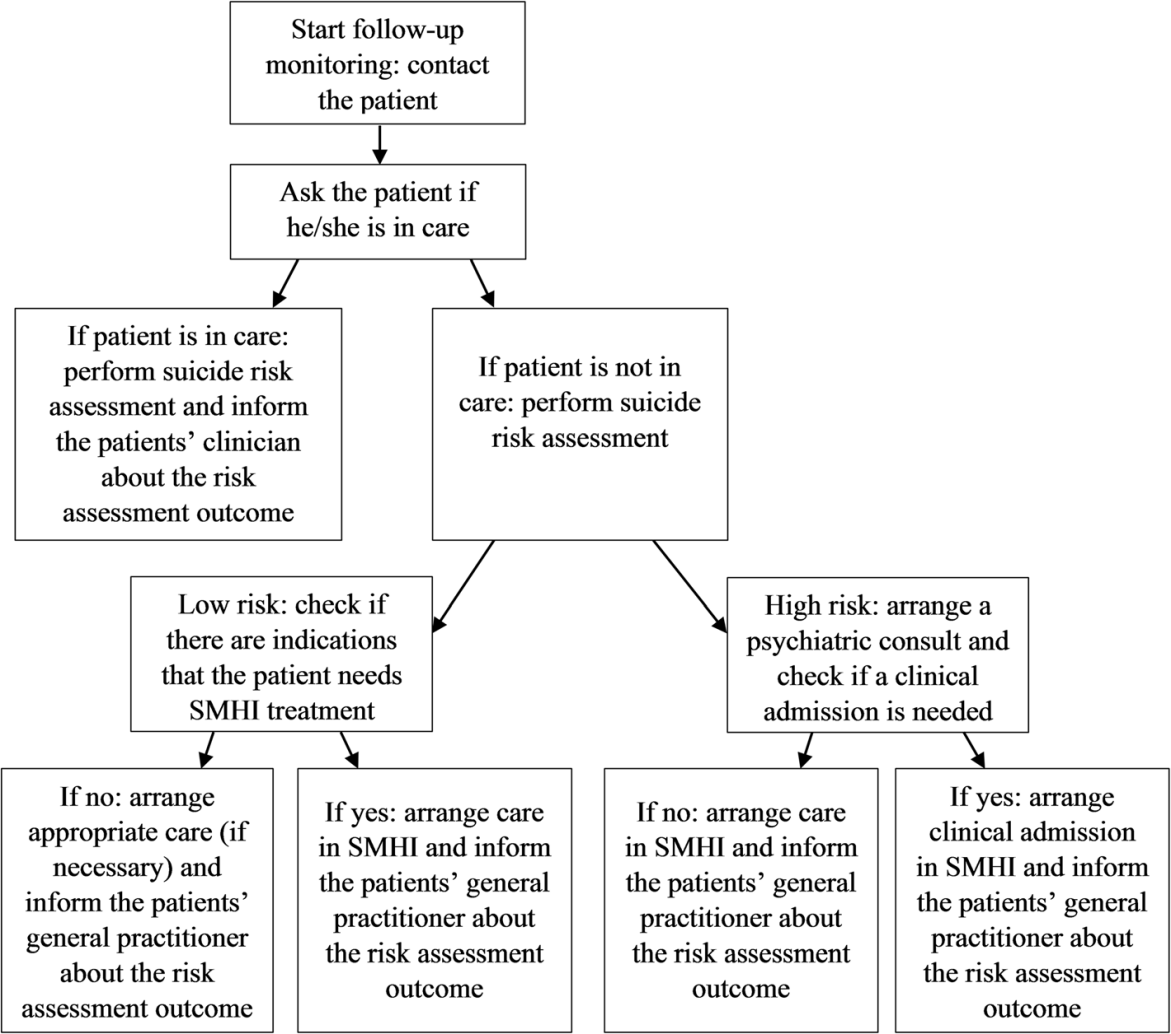
Flowchart of the follow-up monitoring

Telephone follow-up monitoring will be systematically provided for one-year to monitor suicide risk and the continuity of care by the SMHI professional in close collaboration with psychiatrists. The monitoring contacts take place at five fixed times, i.e. 6 weeks after registration in the monitoring system, and after 3, 6, 9 and 12 months. In the monitoring contacts, the suicide risk will be assessed and the person will be asked if he/she is receiving SMHI care. If the person *is* receiving SMHI care, and the suicide risk is low, their clinician will be informed by mail about the suicide risk assessment or the person will be advised to inform their clinician him/herself. In case of a high suicide risk, the clinician will be immediately informed by phone. If the person is *not* receiving SMHI care, and the suicide risk is low, their general practitioner will be informed about the suicide risk assessment. If the suicide risk is high, the crisis care manager will again arrange swift access to appropriate care. A flowchart of follow-up monitoring is provided in Fig. 3.

### Setting

The intervention will be implemented in Noord-Brabant, one of the twelve provinces of the Netherlands. Noord-Brabant comprises an area of over 5000 km^2^, about 2.5 million inhabitants and five specialist mental health institutions. Institutions can participate in the intervention if they signal residents of Noord-Brabant at risk for suicide. We will involve as many settings as possible that can identify people at risk for suicide [39]. Therefore, we do not only include mental health care institutions but organizations in all other settings that might participate by signaling people at risk for suicide. Examples are general hospital emergency rooms and psychiatric departments, general practitioners, occupational physicians, youth mental health care, schools, municipal services, and railway services. All relevant institutions in Noord-Brabant will be approached for participation by the researchers, but when interested, institutions can also contact the researchers themselves. New chain partners may be identified and included during the project. A list of participating organizations can be obtained via the authors.

### Step-wise implementation

The intervention will be stepwise implemented in five subsequent subregions of the province of Noord-Brabant until eventually the system is implemented in the whole province. In the time periods and subregions in which the region is not allocated to the intervention conditions, care as usual is provided. Further details about the stepwise implementation are discussed in paragraph ‘Study design’.

### Scientific evaluation

#### Hypotheses

It is hypothesized that SUPREMOCOL will lead to a reduction of completed suicides of 20% in the province of Noord-Brabant, both in time and compared to the other provinces. Moreover, it is hypothesized that SUPREMOCOL will lead to less completed suicides and suicide attempts, both in association with the stepwise implementation of the system intervention in the subregions of the province, and will diminish suicidal ideation in people registered in the monitoring system.

#### Study design

The design of the SUPREMOCOL study comprises three elements: 1) a stepped wedge trial design, 2) a public health impact evaluation by the RE-AIM framework and 3) an intervention integrity evaluation.

### Stepped wedge trial design

**Fig. 4:**
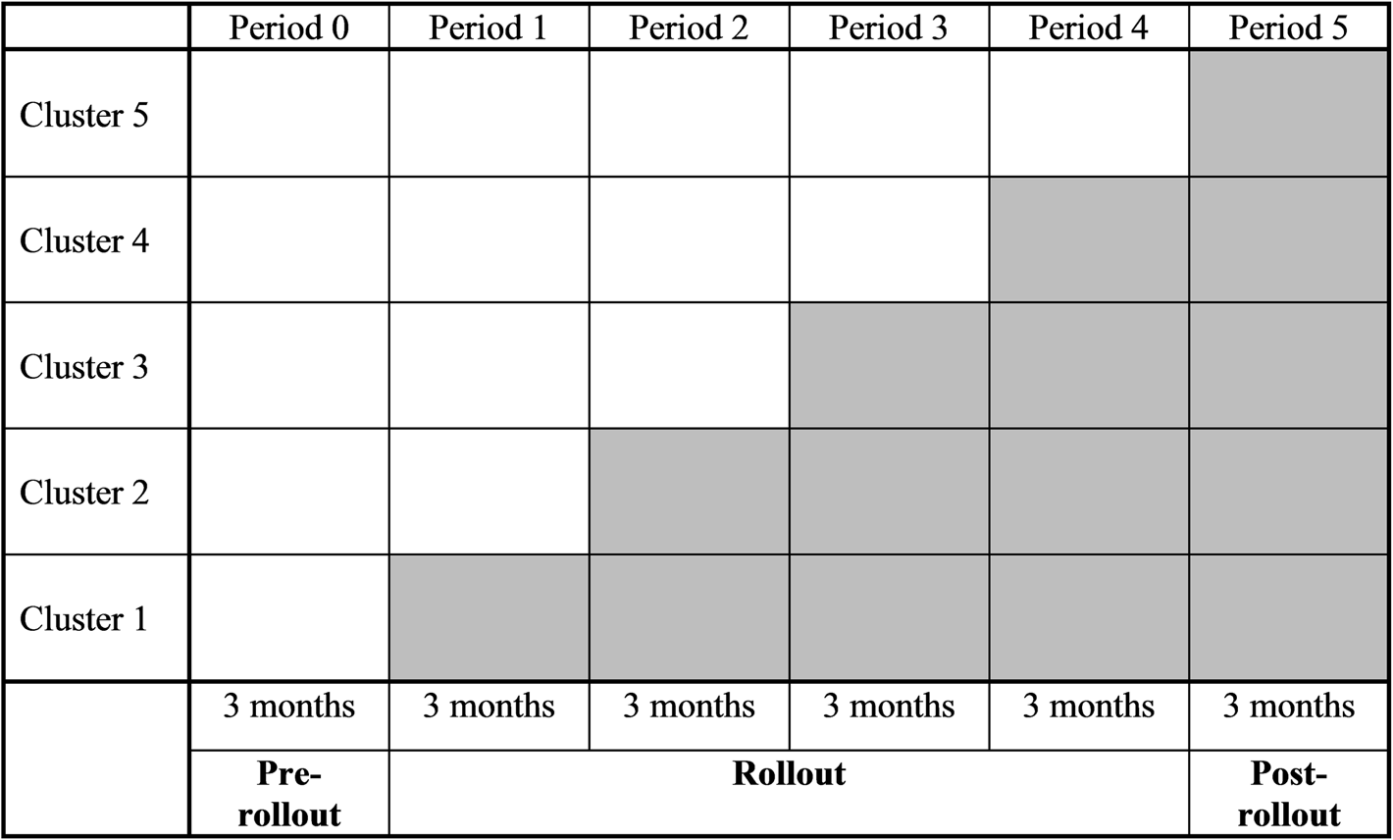
Sequential rollout of the suicide prevention system (shaded cells) to the clusters

The SUPREMOCOL intervention will be evaluated in a stepped wedge trial design (SWTD). In a SWTD, an intervention is sequentially rolled-out to a group of clusters. A cluster is a group of participants that operate in the same geographical area. In our study, a cluster is a specialist mental health care institution together with a group of participating general practitioners, hospitals, and other organisations that operate in that particular SMHI area. We chose a SWTD for ethical, scientific and practical reasons, as further discussed in the ‘Discussion’ of this paper. Stepped wedge trials comprise three main phases: the pre-rollout period, the rollout period and the post-rollout period [40, 41]. In period 0, the so-called pre-rollout period, the intervention has not been implemented in any of the clusters. Next, there will be a rollout period during which clusters are crossing over from the control condition (in this study care as usual) to the intervention (SUPREMOCOL). In period 1, the intervention will be implemented in cluster 1, while the other clusters will keep providing care as usual. In period 2, the intervention will also be implemented in cluster 2 (cluster 1 will still be included), while the other clusters will keep providing care as usual, and so on. In the post-rollout period, the intervention will be implemented in all clusters. A schematic representation of the present SUPREMOCOL trial is given in Fig. 4. Each cell represents a time period and a certain cluster and includes a data collection point. Blank cells represent control periods and shaded cells represent intervention periods [41, 42].

In our SWTD, the implementation of SUPREMOCOL is sequentially rolled-out to five clusters, until eventually the system is implemented in the whole province. For this purpose, the province is divided into five sub-areas that are based on the service areas of the five participating SMHI’s. The SMHI and a few participating organizations are exposed to the experimental condition from the start of their cluster, but at various time points, more organizations in that cluster may participate if they sign up for participation [40].

### Public health impact evaluation

The public health impact of the SUPREMOCOL intervention will be evaluated, as previous studies found that it is important to not only assess the efficacy of an intervention but to also evaluate its impact [43]. For example, an intervention might be very successful in an optimal and controlled experimental condition but might have poor implementation outcomes in complex real-world settings [43]. As a result, many interventions that were found to be effective were never widely adopted in practice [44]. The evaluation to the public health impact of SUPREMOCOL will be done in terms of the five dimensions - Reach, Efficacy, Adoption, Implementation, and Maintenance - of the RE-AIM framework [43]. *Reach* refers to the extent of participation, on an individual level. It can be measured by calculating the rate of the numerator (participants) versus the denominator (population). Additionally, the representativeness of the participants can be assessed by evaluating their characteristics [43]. In this study, reach is the level in which SUPREMOCOL is used by the target population (both patients and professionals) and to their characteristics. *Efficacy* refers to the positive and negative consequences of the intervention and to behavioural and participant satisfaction outcomes [43]. In this study, changes in the outcome measures (suicidal behaviour), compliance with intervention procedures and experiences of patients and professionals with the system were measured to evaluate the efficacy of SUPREMOCOL. The proportion and representativeness of the settings that participate refer to the dimension *adoption* [43]. The level in which settings and professionals are willing to initiate the system, including their characteristics, are examined to measure this dimension in this study. *Implementation* is an individual- and intervention-level dimension and refers to the extent to which an intervention is delivered as intended [43]. In this study, implementation is defined as the level in which the system is implemented according to the protocol. The last dimension is *Maintenance*, which refers to the extent to which the intervention is part of a long-term behavioural change in individuals and organisations and has reached a stable and enduring phase [43]. Maintenance is in this study examined by an evaluation of the long-term compliance with intervention procedures and by attrition analyses.

### Intervention integrity evaluation

Intervention integrity will be measured during implementation. This will be done to gain insight into 1) the extent to which the four pillars of the SUPREMOCOL protocol are implemented in clinical practice as intended, 2) the proportion of settings and practices that adopted the SUPREMOCOL study into their clinical practice, and 3) the proportion of the target population that participated in the SUPREMOCOL study.

### Allocation

If possible, block-wise randomisation will be performed to determine the starting order of the SMHI areas. The ‘blocks’ of areas will be based on the SMHI’s level of implementation and organization, as the implementation of the intervention requires a lot of preparation from the SMHI’s. The block-wise randomization will take place in three steps. First, the SMHI will receive a proposal regarding the implementation of the intervention. Six criteria indicate the extent to which the SMHI is ready for the implementation of SUPREMOCOL. The six criteria are that the SMHI: 1) has a contact person that will be involved in preparing the SMHI for implementing the intervention, 2) provides permission from the board to start the implementation, 3) has set up a team that will provide the daily check for new enrolments in the monitoring system, including providing a first contact with the person, 4) has set up a team that will provide monitoring contacts to the people registered, 5) has received the instructions for working with the monitoring system, and 6) has tested the monitoring system. Second, three blocks will be created, based on the organizations’ level of implementation and organization. Therefore, the three blocks will be: ‘replies to the proposal and is ready to start with the implementation’ (group a), ‘replies to the proposal and is not yet ready to start with the implementation’ (group b) and ‘does not reply to the proposal’ (group c). Third, randomization will take place within the SMHI’s which belong to block a. In this manner, the remaining organizations have extra time to prepare for implementation. If there is only one SMHI in a group, the SMHI will be randomized with a dummy. The districts will be randomized by an independent statistician using a computer algorithm for randomization.

### Outcomes

The primary outcome of this study is completed suicides and the secondary outcome is suicide attempts. Suicidal ideation will, if possible, be an exploratory process measure. Moreover, the impact of the intervention and intervention integrity will be measured. The outcomes and their data collection methods are discussed below.

### Primary outcome: completed suicides

The primary outcome of this study is completed suicides. In the Netherlands, the occurrence of completed suicide is registered by coroners in the ORION system. The records from the ORION system regarding Noord-Brabant will be obtained from the Regional Public Health Institutes (PHI [in Dutch: Gemeentelijke Gezondheidsdienst; GGD]): GGD Brabant-Zuidoost, GGD West-Brabant, and GGD Hart voor Brabant. The PHI’s report their records to Statistics Netherlands, who collect the records for the whole country and make corrections if needed. To evaluate whether SUPREMOCOL leads to a reduction of completed suicides of 20% in the province of Noord-Brabant (hypothesis 1), completed suicides will be assessed during a one-year pre-post measurement for the total population of the province of Noord-Brabant. The pre-measurement will take place in the year before any of the areas in Noord-Brabant have entered the roll-out period (thus: 1 year before the start of period 1 of the SWT; therefore including period 0 of the SWT), and the post-measurement will take place in the year after implementation in all areas (thus: after period 5 of the SWT). National records from Statistics Netherlands will also be used for the pre-post evaluation, as completed suicide rates will be compared in time (pre-post) and per region (Noord-Brabant versus other provinces in the Netherlands). As it is also hypothesized that SUPREMOCOL leads to a reduction of completed suicides and suicide attempts in association with the stepwise implementation of the system intervention in the subregions of the province (hypothesis 2), completed suicides will also be assessed during the stepped wedge trial in the subregions of Noord-Brabant. Regional PHI records will be used for the SWT evaluation. For this, the total number of completed suicides per month will be examined.

### Secondary outcome: Suicide attempts

As suicide attempts and suicidal ideation are among the strongest predictors of completed suicide [1, 12], these two measurements are taken into account in the analysis as well. To evaluate whether SUPREMOCOL leads to a reduction in suicide attempts in association with the stepwise implementation of the system intervention in the subregions of the province (hypothesis 2), the secondary outcome is the rate of attempted suicides in the subregions of Noord-Brabant. This will be evaluated during the stepped wedge trial. The number of suicide attempts will be estimated based on two measurements: 1) the number of ambulance rides that took place after a suicide attempt, which will be provided by the PHI (GGD Brabant-Zuidoost; Ambulancezorg Zuidoost-Brabant) and the regional ambulance transport (Regionaal Ambulancevervoer Brabant Midden-West-Noord), and 2) on the basis of police registrations as provided by the police. The police registrations comprise E14 reports, which are records of incidents for which the police arrived due to an “attempted suicide”.

### Exploratory process measure: suicidal ideation

To evaluate whether SUPREMOCOL leads to a reduction in suicidal ideation in people registered in the monitoring system, if possible, suicidal ideation will be measured by PHQ-9 and SIDAS questionnaires via an online survey. This will be measured at baseline and after 3, 6, 9 and 12 months. Suicidal ideation will also be measured by the PHQ-9 and decision aid outcomes as filled out in the monitoring system at registration, and during the monitoring contacts at 6 weeks, and 3, 6, 9 and 12 months after registration. For the latter, no extra actions from the person are requested as these questionnaires are already filled out for regular care. The Patient Health Questionnaire-9 (PHQ-9) is a reliable and valid clinical and research tool for the measurement of depression severity [26]. The Suicidal Ideation Attributes Scale (SIDAS) is a valid measure for severity of suicidal ideation [45]. Questionnaires will be as short as possible to promote participant retention and complete follow-up; the PHQ-9 questionnaire consists of nine questions and the SIDAS of five questions.

### Exploratory process measure: suicidal behaviour in SMHI patients

Suicidal behaviour in SMHI patients will be measured and taken into account in the analysis of the stepped wegde trial. It will be measured by SMHI records about the number of completed suicides among their patients and admissions into their institution due to suicide risk. The measurement period will take place during the stepped wedge trial and will be collected for each SMHI.

### Public health impact

The impact of the intervention will be evaluated in terms of the five dimensions - Reach, Efficacy, Adoption, Implementation, and Maintenance - of the RE-AIM framework [43]. All dimensions will be evaluated for each of the four pillars of SUPREMOCOL, which are 1) developing and implementing a monitoring system with decision aid to support professionals in reporting, assessing and monitoring people at risk for suicide, 2) providing swift access to people registered in the monitoring system by specialist crisis teams of SMHIs, 3) positioning of nurse care managers to collaborate with psychiatrists in assessment, case management, and guidance to treatment according to the collaborative care model, and 4) providing telephone monitoring at five fixed times during 1 year to people registered in the monitoring system to enhance adherence to treatment. The RE-AIM dimensions and the variables for the SUPREMOCOL study are presented in Table 2.

**Table 2:** Dimensions and definitions of the RE-AIM framework with variables and data sources

Dimension & definition	SUPREMOCOL pillar	Variable	Data source
Reach			
“Proportion of the target population that participated in the intervention” [43]	Pillar 1: monitoring system	• The number (+ characteristics) of chain partners that collaborate in the system versus the number of chain partners that have been approached for collaboration	• Monitoring system • Project group logs
		• The number (+ characteristics) of SMHI’s that collaborate in the system versus the number of SMHI’s that have been approached for collaboration	• Monitoring system • Project group logs
	Pillar 2: swift access	• The number of SMHI’s which are considering swift access versus the total number of participating SMHI’s	• Project group logs • SMHI
	Pillar 3: collaborative care	• The number of SMHI’s which are considering to work according to the collaborative care model versus the total number of participating SMHI’s	• Project group logs • SMHI
	Pillar 4: 12 months follow up	• The number of SMHI’s which are considering to provide 12 months follow up versus the total number of participating SMHI’s	• Monitoring system • SMHI
Efficacy			
“Success rate if implemented as in guidelines; defined as positive outcomes minus negative outcomes” [43]	Pillar 1: monitoring system Pillar 2: swift access Pillar 3: collaborative care Pillar 4: 12 months follow up	• Changes (both positive and negative) in primary and secondary outcome measures (suicidal behaviour), as described in the Methods section	• National and regional health records • PHQ-9 • SIDAS • Monitoring system
		• Experiences of patients and professionals regarding facilitating or hindering factors, as described in the Methods section	• Online surveys • Regular meetings
Adoption			
“Proportion of settings and practices that will adopt this intervention” [43]	Pillar 1: monitoring system	• The number (+ characteristics) of chain partners that made use of the monitoring system versus the number of chain partners who have received an account to this system	• Monitoring system
		• The number (+ characteristics) of SMHI professionals that made use of the monitoring system versus the number of SMHI professionals who have received an account to this system	• Monitoring system
	Pillar 2: swift access	• The number of SMHI departments that have the intention to provide swift access versus the total number of participating SMHI departments	• SMHI
	Pillar 3: collaborative care	• The number of SMHI nurses that have the intention to work according to the collaborative care model versus the total number of SMHI nurses in the SMHI treatment departments	• SMHI
	Pillar 4: 12 months follow up	• The number of SMHI professionals that have the intention to provide follow up contacts versus the total number of SMHI professionals who received an account to the monitoring system	• SMHI
Implementation & Maintenance			
Implementation “Extent to which the intervention is implemented as intended in the real world” [43] Maintenance “Extent to which a program is sustained over time” [43] *Implementation and Maintenance comprise the same variables, however, Maintenance will be measured at nine months.*	Pillar 1: monitoring system	• The number of successful registrations in the monitoring system versus the total number of people attempting suicide (and were in need of an ambulance ride)	• Monitoring system • Ambulance records
		• The number of first contacts that actually took place versus the number of eligible persons for a first contact	• Monitoring system
		• The number of days, after the day on which the first contact should have taken place, the SMHI professional tried to contact the registered person	• Monitoring system
		• The number of days, after the day on which the first contact should have taken place, the first contact between the registered person and the SMHI professional actually took place	• Monitoring system
	Pillar 2: swift access	• The number of crisis assessments or psychiatric consults that were provided by the SMHI professionals versus the total number of crisis assessments or psychiatric consults that were indicated	• Monitoring system
		• The number of swift accesses to care that were arranged by the SMHI professionals versus the total number of swift accessses that were indicated	• Monitoring system
		• The number of clinical admissions that were arranged by the SMHI professionals versus the total number of admissions that were indicated	• Monitoring system
		• The number of people that was referred to their general practitioner by the SMHI professionals versus the total number of people that needed to be referred to their general practitioner	• Monitoring system
	Pillar 3: collaborative care	• The number of SMHI professionals that are involved as case managers versus the total SMHI professionals that have an account to the monitoring system	• Project group logs
		• The number of contacts with the general practitioner by the SMHI professionals versus the total of contacts with the general practitioner that were indicated	• Monitoring system
		• The number of contacts with the psychiatrist by the SMHI professionals versus the total of contacts with the psychiatrist that were indicated	• Monitoring system
	Pillar 4: 12 months follow up	• The number of eligible persons for a follow-up monitoring contact versus the number of follow-up monitoring contacts that actually took place	• Monitoring system
		• The number of days, after the day on which the follow-up monitoring contact should have taken place, the SMHI professional tried to contact the registered person	• Monitoring system
		• The number of days, after the day on which the follow-up monitoring contact should have taken place, the follow-up monitoring contact between the registered person and the SMHI professional actually took place	• Monitoring system

The evaluation of Reach, Efficacy, Adoption, and Implementation will take place during implementation, in the SMHI areas that are allocated to SUPREMOCOL. Evaluation of these domains will be specified per SMHI area and per SWT period (which all last three months). The evaluation of Maintenance will take place nine months after the implementation period, will also last three months and will be evaluated for all SMHI areas separate, as is presented in Table 3. Each dimension is represented on a 0% (no impact) to 100% (most optimal impact) scale. The final Impact score is the function of these five dimensions, calculated for each SMHI region and SWT period [43].

**Table 3:**
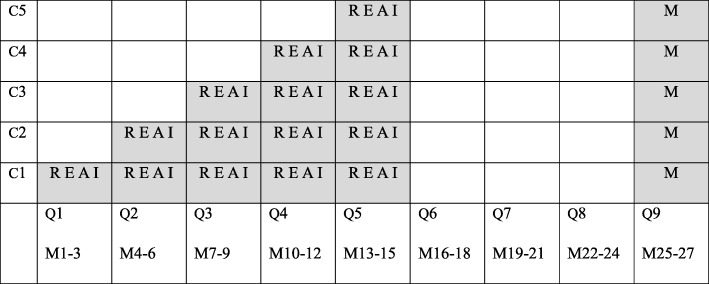
Measurement periods (shaded cells) of the RE-AIM dimensions

Abbreviations: *C*  Cluster, *Q*  quarter of a year, *M*  month, *R*  Reach. *E*  Efficacy, *A*  Adoption, *I*  Implementation, *M*  Maintenance

### Intervention integrity

The integrity of the intervention will be measured during implementation and will be derived from the ‘Reach’, ‘Adoption’ and ‘Implementation’ dimensions of the RE-AIM framework, as can be seen in Table 3. As the integrity of the implementation of the four individual SUPREMOCOL pillars may not equally contribute to the successful implementation of the intervention in total, weight distribution between the four pillars will be applied. The weighing of these four items in the total intervention integrity degree will be determined by the participating SMHI’s and their chain partners of SUPREMOCOL. For this, the study methods for the weighting of fidelity items by stakeholders’ ratings by Oxman and colleagues [46] will be followed. The chain partners will be asked in a meeting to independently rate the relative importance of the variables belonging to the four pillars in successful implementation, by giving a score from 1 to 10 to the four pillars. Mean intervention degree ratings will be calculated, taking into account the weighing of these items.

#### Sample size

The sample size calculation is based on completed suicide rates, as this is the primary outcome of this study. The USA study of which we adapted our systems approach established a drop in potentially salvageable deaths from 34% (20/58) to 15% (9/60) (*p* < .02) following implementation, which is a Hazard Ratio of 2.3. We assume that in trauma patients, compliance of patients with entrance to care, if provided, was 100%, as the trauma is a one-time occurrence and involuntary. In people at risk for suicide, this risk may be sustained for a longer period of time, require multiple entrances into care and compliance may not always be the case. Hence, we want to be able to detect a smaller difference, which is of 20% in the completed suicides, as specified in our hypothesis. This corresponds to a rate ratio of 1.25. Based on an alpha of 0.05 (two-sided) and a power of 0.80, for this purpose, the minimal number of events would be 184 in each year. As completed suicides are the primary outcome we do not expect any loss to follow up for that outcome. As in 2017, the number of completed suicides in Noord-Brabant reported by Statistics Netherlands was 365, and all relevant settings in the region will collaborate, we expect that it will be feasible to find a difference of 20% in completed suicides, if such a difference exists, with an alpha of 0.05 and a power of 0.80.

#### Inclusion criteria

People who are registered in the monitoring system will be invited to participate in a survey evaluating suicidal ideation by filling out the PHQ-9 and SIDAS questionnaires. Inclusion criteria for participation in this survey are that participants are 1) 18 years or older and 2) Dutch-speaking. They will be asked for written informed consent, as further discussed in paragraph ‘Ethics approval and consent to participate’.

#### Statistical methods

To examine whether SUPREMOCOL leads to a reduction of completed suicides of 20% in the province of Noord-Brabant, the total number of completed suicides in the post-measurement will be compared to the total number of completed suicides in the pre-measurement. As the total number of residents in this province might have changed, we will also compare the pre- and post-incidence rates of suicides. An exact rate ratio test will be used. To take general trends over time into account, we will compare the incidence ratio in Noord-Brabant with the incidence ratios in other provinces in Noord-Brabant, and with the other provinces combined.

To examine whether SUPREMOCOL leads to a reduction of completed suicides and suicide attempts in association with the stepwise implementation of the system intervention in the subregions of the province, the total number of completed suicides and suicide attempts in each month will be compared to the two levels of our stepped wedge trial: region and time. A poison multilevel model will be used. The occurrence of completed suicide or admissions due to suicide risk in an SMHI will also be taken into account in the analysis. The suicide rates will be corrected for seasonal fluctuations [45]. In a second model, organisational differences between the regions, such as available number of crisis departments, will be taken into account. In a third model, the level of treatment integrity per subregion will be taken into account as well.

To explore whether SUPREMOCOL leads to a reduction of suicidal ideation in people registered in the monitoring system, the scores on the PHQ-9, SIDAS, and decision aid -that will be filled out by participants during the year that they receive the Supremocol additional care- will be compared by repeated measures. This will be used as exploratory process evaluation, as there is no control group for this.

To examine the impact of SUPREMOCOL with regards to reach, efficacy, adoption, implementation, and maintenance, an impact score will be calculated for each SMHI region and time period. This scores will be based on the function of all five RE-AIM dimensions, which are represented on a 0% (no impact) to 100% (best possible impact) scale [43].

### Safety and confidentially

#### Harms

In general, the risk of including suicidal patients in research studies is expected to be low. Huisman and Kerkhof (2017) have studied the effects of inclusion of suicidal participants in scientific research and questioning suicidality for scientific research. They found that including suicidal people in research trials does not lead to an increase in completed suicides or suicide attempts [47]. Furthermore, it has to be stressed that our stepped wedge trial includes a design that has its effect at the organisational level, not at the patient level. Hence, people always receive care as usual. In case a completed suicide occurs in a person that is registered in the SUPREMOCOL monitoring system, the suicide will be reported to the Medical Ethics Committee, the sponsor (GGz Breburg), and the funder (Netherlands Organisation for Health Research and Development) of this study. A careful and detailed assessment will be made as to whether there is a link between the suicide and the registration in the monitoring system. Depending on the outcome, an advice is given to the parties to end or continue the trial. In case the Health Care Inspection would prematurely terminate the study, the participating organizations and participants will be fully informed.

#### Ancillary and post-trial care

In accordance to section 10, subsection 4, of the WMO, the sponsor of this study, GGz Breburg, will suspend the study if there is sufficient ground that continuation of the study will jeopardize the health or safety of patients. GGz Breburg will notify the Medical Ethics Committee Brabant without undue delay of a temporary halt including the reason for such an action. The study will be suspended pending a further positive decision by the Medical Ethics Committee Brabant. The investigator will take care that all participants are kept informed. In addition, all (serious) adverse events will be reported and followed until they have abated, or until a stable situation has been reached. Depending on the event, follow up may require additional tests or medical procedures as indicated, and/or referral to the general physician or a medical specialist.

#### Confidentiality

The monitoring system will be a web-based system, encrypted and build on a secured server. A person can only be registered in this monitoring system if he or she provides permission for registration. Participants’ data will be handled confidentially and in accordance with the Dutch Personal Data Protection Act. A subject identification code list will be used to link the data to a participant. The data of the participating participants are encrypted and stored under a number that is not traceable to their identity. Only the researchers will have access to the key of the encryption, which will be stored separately from the data set. In the publications, the results cannot be traced to individual participants. The key to the code will be safeguarded by the researcher, in case the data will be kept for a longer period of time.

## Discussion

In this paper, we aim to outline the development and design of the SUPREMOCOL study. Implementation and evaluation of this intervention will be performed by a stepped wedge trial design and the RE-AIM framework will be used to evaluate the impact of the intervention. Several potential strengths and limitations of this study can be expected.

### Potential strengths of the study

This study has three major strengths. First, the stepped wedge trial design combined with the RE-AIM framework is a strength, as it offers multiple benefits for implementation and evaluation. In our opinion, the SWTD is the most ethical design, as all areas act as their own control group, and therefore no areas will be excluded from the suicide prevention system [41], as we believe SUPREMOCOL will have considerable added value to the current care as usual [41, 48]. A stepwise implementation of the intervention is also highly desirable due to scientific and practical reasons [41, 48], as it creates the possibility to control for a time effect by enabling comparison between the old and new organization of health care at the sub-levels of the clusters. Furthermore, implementing the suicide prevention system on a provincial-wide scale at once would be rather impractical and a threat to the feasibility of the project. This stepwise implementation will make the implementation more manageable. The combination with the RE-AIM framework is to our opinion a valuable addition to the SWTD, as it will provide insight into the impact of the intervention. This information is useful in the evaluation of the efficacy of SUPREMOCOL. Moreover, information from the perspective of the stakeholders and about facilitating and hindering factors in implementation could be used to further improve the prevention system.

A second major strength will be that we choose to evaluate the efficacy of the intervention with the outcome parameters completed suicides, suicide attempts, and suicide ideation. This gives direct information about the effectiveness of the intervention, as preventing suicide is the ultimate goal of suicide prevention.

Third, it has been argued that effective action towards reducing suicide would need combined interventions by different providers in multiple domains [29, 30, 49, 50] – so-called multilevel interventions [1, 51]. Therefore, we will not only include (mental) health care institutions but organizations in all other settings that can participate by signaling and registering people at risk for suicide in the monitoring system, which is also considered as a strength of this study [39].

### Potential limitations of the study

We identified two factors that may form potential limitations of our study. A first potential limitation is that randomization of the starting order of the clusters will not be feasible as the SMHI’s might be in different phases in terms of preparation for implementing the intervention. We identified this potential limitation as there are, for example, changes and shortages in personnel within the crisis care departments of the SMHI’s, which will make the implementation of the intervention a challenge. However, as discussed in previous research, although randomization in stepped wedge trial designs is recommended, the trial might also be performed without, if it is logistically not feasible [52]. Given the vulnerability of our target group, we will not assign clusters to the intervention condition if they are not completely ready, and intervention allocation to the SMHI areas will only be determined based on six criteria indicating the readiness of the SMHI for the implementation.

A second potential limitation is that SUPREMOCOL is not the only suicide prevention initiative that residents in Noord-Brabant are exposed to. For example, as gatekeepers training has gained increased attention in the Netherlands, (mental) health care professionals and other participating chain partners might have increasing awareness to and better screening skills for screening suicide risk. This might be beneficial for the clusters that start the implementation of SUPREMOCOL on a later moment. Another example is that some of the participating organizations operate in the whole province, such as the railway services. In these organizations, the same professionals will participate during the whole duration of our trial, only the geographical areas that are exposed to the intervention will expand. It is possible that they will be better trained in delivering the intervention in the last period of the SWTD, in comparison to period one, which is also advantageous for clusters that start on a later moment. Although this might be beneficial for our outcomes, it might also bias our results.

### Contribution of the study

This study has the potential to provide a new effective suicide prevention intervention for people with a high to moderate suicide risk as well as to improve the chain of care. This study will provide valuable information with regard to the effectiveness of the regional suicide prevention system SUPREMOCOL. Furthermore, the will create insight into the impact, facilitating and hindering factors of its implementation. The findings may give an evidence base for further dissemination of regional suicide prevention systems aiming to reduce completed suicides, suicide attempts and suicide ideation among citizens.

## Data Availability

Research data will be stored at Tilburg University and comply with the quality infrastructure of Tilburg University. Data are managed and monitored with the required accuracy and organizational and technical measures to protect the processing of data has been taken. The research group, management board and Tranzo’s (Tilburg University) quality professional will conduct quality checks on the data during the project to check if they are complete, correct and consistent. The research group will apply the recommended retention period for the data of at least 15 years. After completion of the project, Tilburg University will have governance over the final trial dataset, in compliance with their quality infrastructure. A Data Monitoring Committee was not needed in this study as all suicides that occur in the SMHI will be reported to the medical director of the SMHI and this will be controlled by the Health Care Inspection.

## Abbreviations

MGSB: Multidisciplinary Guideline Diagnostics and Treatment of Suicidal Behaviour
GDPR: European Union General Data Protection Regulation
PHQ-9: Patient Health Questionnaire-9
RE-AIM: Reach, Efficacy, Adoption, Implementation, Maintenance
SIDAS: Suicidal Ideation Attributes Scale
SMHI: Specialist Mental Healthcare Institution
SUPREMOCOL: Suicide Prevention by Monitoring and Collaborative Care
SWTD: Stepped Wedge Trial Design
